# Non-Uniformly Powered and Spaced Corporate Feeding Power Divider for High-Gain Beam with Low SLL in Millimeter-Wave Antenna Array

**DOI:** 10.3390/s20174753

**Published:** 2020-08-22

**Authors:** Md Nazim Uddin, Sangjo Choi

**Affiliations:** Department of Electrical Engineering, University of Ulsan, Ulsan 44610, Korea; 20195162@mail.ulsan.ac.kr

**Keywords:** corporate feeding, parasitic patch, sidelobe level, millimeter wave antenna array

## Abstract

A corporate feeding antenna array with parasitic patches has been investigated previously for millimeter-wave applications due to its high gain and wide bandwidth. However, the parasitic patch integration in the uniformly powered and spaced patch antenna array led to a high sidelobe level (SLL). In this study, we designed a non-uniformly powered and spaced corporate feeding network to feed a 12-element parasitic patch-integrated microstrip antenna array for SLL reduction at 28 GHz in the millimeter-wave band. In the power divider, we arranged two one-to-six unequally feeding power dividers from the opposite side to feed 12 antenna elements with non-uniform excitation, and effectively controlled the spacing between antenna elements. The two opposite input ports from the power divider were fed 180° out-of-phase for good isolation between the adjacent antenna elements. To verify the SLL reduction effect from the non-uniform spacing in the array, we designed two non-uniformly powered patch antenna arrays with uniform and non-uniform spacing. In the measurement, the non-uniformly powered and spaced patch antenna array demonstrated a nearly 16.56 dBi boresight gain and −17.27 dB SLL, which is nearly 2 dB lower than the uniformly spaced counterpart. Finally, we expect that the non-uniformly powered and spaced high gain patch antenna array with a low SLL will be suitable for millimeter-wave communication applications.

## 1. Introduction

Millimeter-wave band systems have been actively studied for fifth-generation (5G) smartphones, which will provide higher data rates and capacity [1,2,3]. However, the millimeter-wave experiences high path loss; therefore, 5G-related hardware needs to increase the gain of the transmitter and receiver systems [4,5,6]. With a limited power resource, a directive antenna system that focuses energy in a specific direction with a sharp pencil beam is considered key hardware in millimeter-wave applications. The sharp beam can be realized by a high-gain antenna with a low sidelobe level (SLL) that requires an array of antenna elements.

In a patch antenna array, a popular antenna topology for millimeter-wave systems, parasitic patches have been implemented on a feeding patch array with a spacer to further increase the antenna gain [7,8,9]. Despite the increased gain from the parasitic patches, a main-beam offset in the E plane due to the near-field coupling between the parasitic and feeding patches was identified, and out-of-phase feeding to adjacent patches in the opposite feeding position was used as a solution [10]. In [10], an antenna array with 16 patches achieved 19.88 dBi gain, with the maximum beam at the boresight near 28 GHz; however, the array did not utilize methods to reduce SLL, and the value was limited to −13.4 dB. The other dipole, patch and aperture antenna arrays for 5G millimeter-wave applications also showed limited SLL values near −10 dB to −15 dB [11,12,13,14,15].

For SLL reduction in the antenna array, antenna elements can be powered in a non-uniform fashion following binomial or Chebyshev distribution [16] (pp. 323–325). A straightforward way to control the feeding amplitude is the tapering of the sizes of radiating elements, i.e., patches and apertures in the array [17,18]. Instead, corporate feed power dividers with unequal power split junctions were used [19,20,21,22,23,24,25]. In a series-fed patch array, the characteristic impedance of the feeding microstrip lines was tuned [19]. The power dividers with an unequal power division ratio in the T-junctions of the substrate integrated waveguide (SIW) are commonly used in the millimeter-wave band for tapering the excitation [20,21,22,23,24,25]. Additionally, the number of power dividing junctions from the input to each output (the level of the power split) in the microstrip line and SIW was controlled for tapering amplitude [25,26]. The performance of this power divider type mainly depends on the power split level for each output; therefore, the power divider’s design process can be simple, and its sensitivity to fabrication uncertainty can be low.

Recently, a non-uniformly spaced corporate feed network was also introduced as a solution to further reduce the SLL of a patch antenna array [27,28,29,30]. In [27,28], non-uniformly spaced series-fed arrays were introduced to reduce SLL at 9 GHz and 24 GHz, respectively, but these arrays also need to taper the radiating elements. A non-uniformly spaced elliptical array of 8, 12 and 20 elements with uniform excitation was designed, and nearly −20 dB SLL was verified in the numerical simulations [29]. In [30], 12 patch elements, fed by a non-uniformly spaced power divider with amplitude tapering using different levels of power split in each output, achieved nearly −20 dB SLL at 5.8 GHz. However, the result was only verified in the numerical simulation, and a microstrip line-based power divider with non-uniform spacing is yet to be designed in the millimeter-wave band, and its SLL reduction has not been verified experimentally.

In this paper, we present a non-uniformly spaced microstrip line-based power divider to feed 12 patch antennas with amplitude tapering using different levels of T-junctions, and demonstrate the SLL reduction at 28 GHz experimentally. Differently from [30], the feeding network utilized two one-to-six unequally split power dividers, fed from the opposite side with out-of-phase, to maintain the maximum beam at the boresight by reducing mutual coupling among adjacent antenna elements. In addition, the patch antenna implemented the parasitic patch to increase gain for practical applications; therefore, non-uniform spacing for the lowest SLL was determined using full-wave simulations instead of the analytical array factor. Despite the non-uniform spacing between the antenna elements, the power divider should maintain in-phase excitation for all the antenna elements. This design challenge was tackled by the T-junction position and the delay line optimization. As a counterpart to the non-uniformly powered and spaced power divider, we also designed a non-uniformly excited but uniformly spaced power divider for the same number of patches in order to prove SLL reduction from a non-uniformly spaced array effectively. In this paper, we first introduce a single patch antenna with parasitic elements for maximum gain. Then, the design procedure for both non-uniformly powered dividers with uniform and non-uniform spacing will be followed. Finally, we demonstrate the performance of the power dividers by analyzing the gain and SLL values of both antenna arrays in the simulation and measurement.

## 2. Design and Optimization of Antenna Array

### 2.1. Design of Single Patch with Parasitic Element

**Figure 1:**
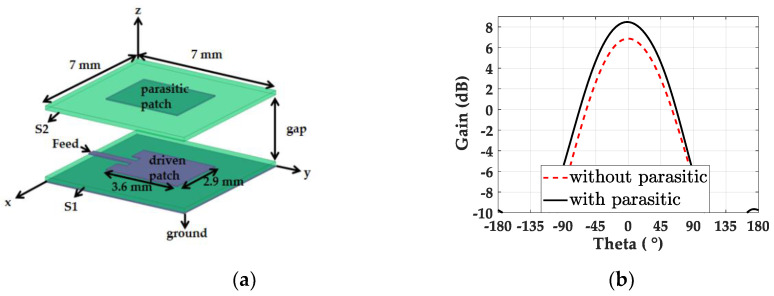
(**a**) Structure of the single patch with the parasitic element. (**b**) Gain of the patch with and without the parasitic patch in the x-z plane along theta (θ).

We designed a single patch antenna with a parasitic patch, as shown in Figure 1a. The parasitic patch mounted on the driven patch realizes resonance coupling, and hence the bandwidth and gain of the design can be improved [31]. In the design, two equally sized 7 × 7 mm^2^ substrates, S_1_ and S_2_, were placed with an air gap of 1 mm to separate the driven and parasitic patch. A thin Rogers 5880 substrate with a thickness of 0.127 mm, dielectric constant of 2.2, and loss tangent of 0.0009 was used for both patches. The driven patch and parasitic patch were each 3.6 × 2.9 mm^2^ in size. On the driven patch, the inset feed length and width were 0.45 mm and 1.1 mm, respectively, for 50 Ω matching. The simulated gain values of the patch antenna on the x-z plane with and without the parasitic patch are presented in Figure 1b, illustrating the 1.63 dB increased gain of the patch antenna at 28 GHz due to the parasitic element. A commercial full-wave simulation tool, the high-frequency structure simulator (HFSS), was used to model the patch antenna and the following power dividers [32].

### 2.2. Effect of Non-Uniform Excitation and Spacing on SLL

We calculated the array factor using the excitation amplitudes and inter-element distances of 12 antenna elements, and found the optimum distances with a given tapered amplitude condition for the lowest SLL. Instead of 8 or 16 elements that opt to be equally powered, the 12-element array was chosen because a corresponding feed network can realize a non-equal output power with different power split levels. The array factor formula for the 12-element linear array is shown in Equation (1).

(1) $$\mathrm{AF}\left(\theta \right)=\overset{6}{\underset{n=-6}{\sum }}a_{n}e^{{\mathrm{jkx}}_{n\;}\sin\left(\theta \right)}$$

**Figure 2:**
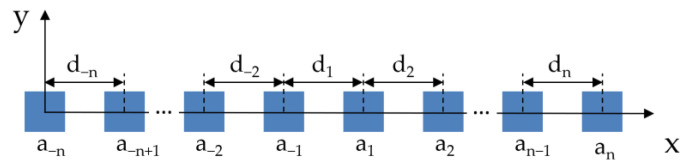
Linear patch array of an even number of elements with the excitation amplitude (a_n_) of the nth antenna element and the distance (d_n_) between n-1th and nth elements on the x–y plane. Excitation and spacing are set by symmetry (a_−n_ = a_n_ and d_−n_ = d_n_).

Here, n, the index for the antenna element, has a range between −6 and 6, with an increment of 1, and n = 0 is excluded. a_n_ is the nth element’s excitation amplitude, x_n_ is the location of the nth element along the *x* axis, k is the wavenumber, and θ is the elevation angle from the *z* axis. x_n_ was determined by d_n_, the distance between n-1th and nth elements, and d_1_ means the distance between two central patches in the array, as shown in Figure 2. For simplicity, we only defined a_n_ and d_n_ for positive n, and negative n cases were set by symmetry using a_−n_ = a_n_ and d_−n_ = d_n_.

**Figure 3:**
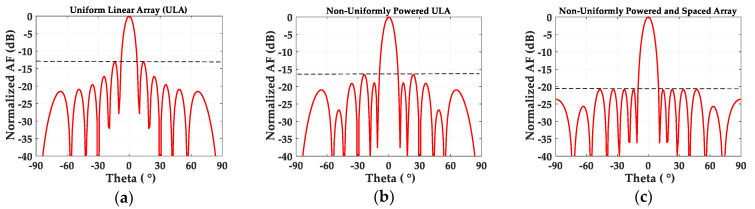
Normalized array factors of the 12-element array with (**a**) uniform amplitudes [1, 1, 1, 1, 1, 1] and uniform λ/2-spacing, (**b**) non-uniform amplitudes [1, 1, 0.707, 0.707, 0.707, 0.707] and uniform λ/2-spacing, and (**c**) non-uniform amplitudes [1, 1, 0.707, 0.707, 0.707, 0.707] and non-uniform spacing [0.459λ, 0.528λ, 0.454λ, 0.421λ, 0.548λ, 0.598λ].

The normalized amplitudes of the six elements for a uniform amplitude array (ULA) were set to be [a_1_, a_2_, …, a_6_] = [1, 1, 1, 1, 1, 1]. For a non-uniformly excited array with a lower SLL, the normalized power levels of two-thirds of the elements were tapered, with [1, 1, 0.5, 0.5, 0.5, 0.5] using one more half-power division for n = 3, 4, 5 and 6. Therefore, the corresponding non-uniform amplitudes were set to be [a_1_, a_2_, …, a_6_] = [1, 1, 0.707, 0.707, 0.707, 0.707]. With this amplitude condition, we searched the distances (d_n_) between the patch elements, which provide the lowest SLL near the half-lambda distance with a +/− 0.1 lambda gap. Finally, non-uniform distances [d_1_, d_2_, …, d_6_] = [0.459λ, 0.528λ, 0.454λ, 0.421λ, 0.548λ, 0.598λ] provided the lowest SLL of –20.90 dB, and the optimum distances show similar values to those previously reported for the 12-element array structure [30]. Figure 3a shows the normalized array factor of the uniformly (0.5λ) spaced ULA of 12 antenna elements along theta (θ), indicating an SLL of −13.07 dB. In Figure 3b, the non-uniform amplitude excitation of [1, 1, 0.707, 0.707, 0.707, 0.707] with uniform spacing (0.5λ) provides a ~3.5 dB lower SLL of −16.55 dB, compared to the ULA case. Finally, Figure 3c demonstrates that a minimum SLL value of −20.90 dB is possible with the non-uniformly powered and spaced antenna array. The array factor calculations verify that the optimum non-uniform distances between the antenna elements further reduce the SLL. In the next sections, the physical realization of power dividers for the 12-element patch array operating at 28 GHz will be presented.

### 2.3. Design of Non-Uniformly Powered and Uniformly Spaced Antenna Array

#### 2.3.1. Non-Uniformly Powered and Uniformly Spaced Power Divider Design

**Figure 4:**
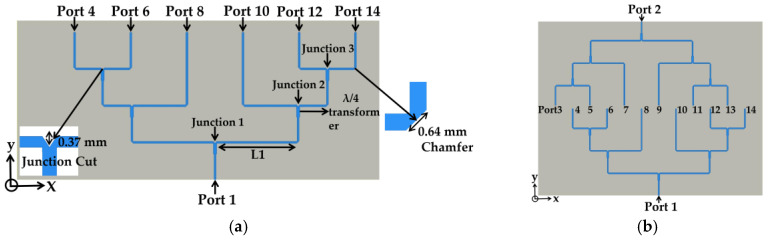
(**a**) one-to-six way non-uniformly powered and uniformly spaced power divider. (**b**) Two one-to-six-way power dividers for 12 feeding ports to the antenna elements.

We designed a six-way power divider, shown in Figure 4a, to realize non-uniform excitation amplitudes of [1, 1, 0.707, 0.707, 0.707, 0.707] in 12 patch elements for SLL reduction. Two one-to-six-way symmetrical power dividers were placed in the opposite direction, and 12 output ports were arranged with a λ/2 distance for uniform spacing. Overall, a 2-to-12 power divider was designed to feed the 12 patch antennas, and two inputs ports were excited with 180° difference to maintain in-phase field excitation in all the antenna elements shown in Figure 4b. In the one-to-six-way power divider, the input and output transmission lines were designed with 50 Ω characteristic impedance (Z_0_), and the quarter wavelength transformers with Z_0_ of 35.35 Ω in the junctions 1, 2 and 3 were used to match the impedance between the one input and two output branches of the junction shown in Figure 4a. Then, the right half of the power divider was mirrored to the left side and a one-to-six power divider design was completed with symmetric form. The spacing between the 12 output ports in Figure 4b was set to λ/2, and the separation between the transformer junctions along the *y* axis was chosen to be greater than half of the guided wavelength for mutual coupling reduction [33]. Overall, ports 8 and 10 receive one-quarter of the input power, and the other output ports receive the half-power of ports 8 and 10. Therefore, ports 8 and 10 support twice the power of ports 4, 6, 12 and 14.

Table 1 shows the normalized ideal excitation for ports 4, 6, 8, 10, 12 and 14, which follow the required non-uniform excitation amplitudes of [1, 1, 0.707, 0.707, 0.707, 0.707] for SLL reduction. The simulated power and excitation values from the same ports in the power divider are shown in Table 2. The simulated values follow the trend as Table 1, with lower magnitudes due to loss and reflection from the bends and the T-junctions. In the power divider design, the reflection from the discontinuities was reduced by chamfering the bending edges and cutting the center of the junction, as shown in Figure 4a.

#### 2.3.2. Phase Correction of Power Divider

**Figure 5:**
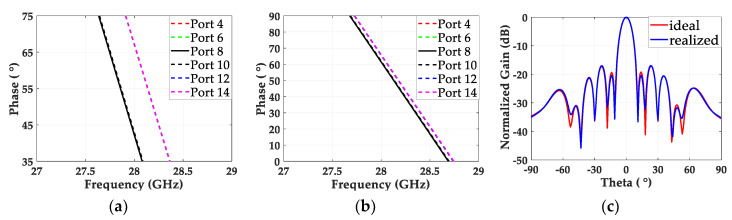
Phase delays from the input port to output ports 4, 6, 8, 10, 12 and 14 when (**a**) L1 = 16.37 mm and (**b**) L1 = 16.11 mm. (**c**) Normalized gain along theta (θ) of the 12 patch elements individually fed by the realized amplitudes and phases from the designed power divider and the ideal values.

In the designed 2-to-12 power divider, the phase delays for all the output ports should be the same in order to maintain in-phase feeding for all the antenna elements. Because different numbers of quarter-wave impedance transformers were used for different output ports, a modification of the power divider was needed. Figure 5a shows that the phase delays from port 1 to ports 4, 6, 12 and 14 are the same, at 67°, which is greater than the 42° of the other signal paths for port 8 and 10 at 28 GHz. To balance the phase delays between two different paths, we tuned the length of L1 in the power divider by moving the position of junction 1 and maintaining the positions of the other junctions. We reduced the initial L1 length of 16.37 mm by 0.27 mm in order to compensate for the 25° phase difference. Then, we further tuned L1 in the full-wave simulation, and achieved almost identical phases with an L1 of 16.11 mm, as shown in Figure 5b. To understand the effect of the output of the optimized power divider on the radiation pattern, we excited the 12 patch elements individually with the realized amplitudes and phases from the power divider, and calculated the normalized gain along theta (θ) in the full-wave simulations. The gain pattern from the array fed by ideal amplitudes and phases was also achieved, and Figure 5c shows that both patterns are almost overlapped, proving that the designed power divider provides broadside radiation with the desired SLL suppression.

#### 2.3.3. Non-Uniformly Powered and Uniformly Spaced Antenna Array

**Figure 6:**
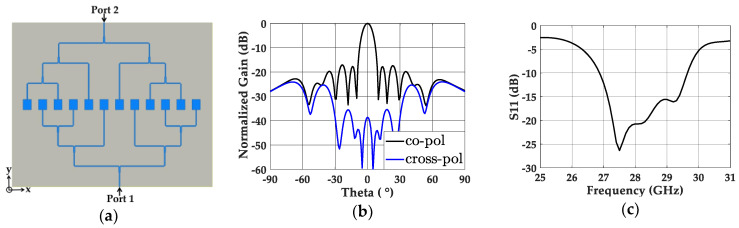
(**a**) Top view of the non-uniformly powered and uniformly spaced 12-element patch array. (**b**) Simulated normalized gain of the non-uniformly powered and uniformly spaced 12-element patch array with co- and cross-polarization. (**c**) Simulated S_11_ of the same antenna array.

We integrated a uniform λ/2-spaced 12-element patch antenna array with the optimized 2-to-12 power divider, as shown in Figure 6a. On top of the patch antenna elements, the parasitic patches were mounted with a 1 mm air gap, and two input ports (port 1 and port 2) were fed with a 180° phase difference. The simulation results showed a 17.12 dB gain on the boresight, and the normalized radiation pattern in Figure 6b indicates an SLL of −16.98 dB on the x–z plane. The level of SLL follows the −16.55 dB SLL of the array factor of the corresponding array, indicating that the required non-uniform power distribution was realized through the designed power divider. The nearly 40 dB lower cross-polarized radiation level compared to the co-polarization also indicates that the radiation from the power divider to the cross-polarization is negligible. Figure 6c shows the wide bandwidth of 9.9% from 26.87 to 29.64 GHz for S_11_ < −10 dB.

### 2.4. Design of Non-Uniformly Spaced Antenna Array

**Figure 7:**
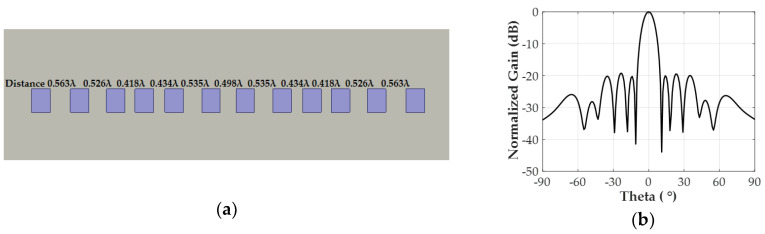
(**a**) Non-uniformly spaced 12-element patch array without a power divider. (**b**) Normalized gain of the non-uniformly powered and non-uniformly spaced patch array without a power divider in the simulation.

To further reduce SLL, we designed a non-uniform inter-element spaced power divider in addition to the non-uniform amplitudes of [1, 1, 0.707, 0.707, 0.707, 0.707]. On top of the non-uniform distances of [0.459λ, 0.528λ, 0.454λ, 0.421λ, 0.548λ, 0.598λ] used in the array factor calculation, we optimized the non-uniform distances using the full-wave simulation to consider the coupling effect of the parasitic patch. The optimum distances for half of the array were [0.498λ, 0.535λ, 0.434λ, 0.418λ, 0.526λ, 0.563λ], and the other side of the array was mirrored, as shown in Figure 7a. The maximum SLL from the non-uniformly powered 12 patch antennas with the optimized distances without a power divider is nearly −19.25 dB, as shown in Figure 7b. According to the optimum inter-element spacing for the lowest SLL, we then designed a one-to-six-way power divider and integrated the 12 patch antennas with two sets of one-to-six power dividers.

#### 2.4.1. Non-Uniformly Powered and Spaced Power Divider Design

**Figure 8:**
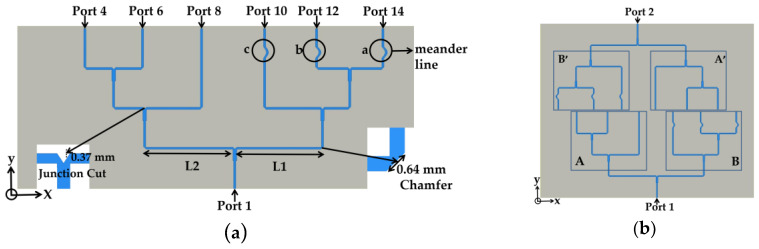

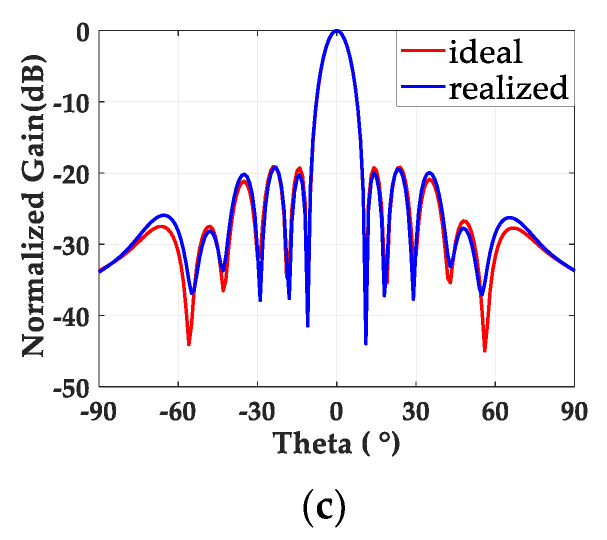
(**a**) One-to-six-way uniformly powered and spaced power divider. (**b**) 2-to-12 non-uniformly powered and spaced divider. (**c**) Normalized gain along theta (θ) of the 12 patch elements individually fed by the realized amplitudes and phases from the designed power divider and the ideal values.

In contrast to the uniformly-spaced power divider, the non-uniformly-spaced structure led to four different signal paths (Port 4 and 6, Port 8, Port 10, and Port 12 and 14) because the power divider lost the symmetry along the *y* axis, as shown in Figure 8a. In the power divider, L1 can tune phase delays for ports 10, 12 and 14, and L2 can tune only the other signal paths. We changed L1 from 15.7921 mm to 15.575 mm, changed L2 from 16.5152 mm to 16.005 mm, and reduced the phase delay between two different groups with 15° difference, as shown in column 2 of Table 3. However, the tuning of L1 and L2 was not enough to excite all of the ports with identical phase delays. 

To further tune the phase delays, we introduced meander lines for ports 10, 12 and 14 in order to tune their physical lengths. Here a, b and c are denoted as the length differences between the straight line and the meander line. The meander lines for ports 12 and 14 increased the paths by 0.38 mm (a = b). The path for port 10 was lengthened by 0.27 mm (c). The added meander lines maintained the maximum phase difference at nearly 5.09°, as shown in column 3 of Table 3. Finally, we designed a 2-to-12 power divider by mounting a one-to-six power divider rotated along the *y* axis from the opposite side. The 12 output ports of two one-to-six power dividers were arranged with [0.498λ, 0.535λ, 0.434λ, 0.418λ, 0.526λ, 0.563λ] distances, as shown in Figure 8b. Here, A and A’ are symmetric, as are B and B’. Before we connected the power divider to the 12 patch antennas, we excited each antenna with the realized amplitudes and phases from the power divider and calculated the normalized gain along theta (θ). Figure 8c proves that the gain pattern from the realized output from the power divider is almost the same as the pattern calculated from the array with the ideal in-phase excitation and amplitude tapering.

#### 2.4.2. Non-Uniformly Powered and Spaced Antenna Array

**Figure 9:**
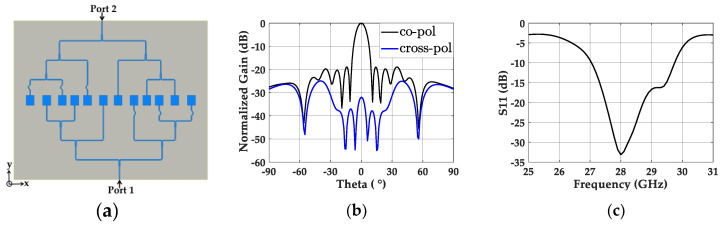
(**a**) Top view of the non-uniformly powered and spaced 12-element patch array. (**b**) Simulated normalized gain of the non-uniformly powered and spaced 12-element patch array with co- and cross-polarization. (**c**) Simulated S_11_ of the same antenna array.

The non-uniformly spaced 2-to-12 power divider was integrated with 12 patch antennas with parasitic elements, as shown in Figure 9a. Here, two input ports were also fed with a 180° phase difference, and two equal-sized substrates (70 × 57.3 mm^2^) for the driven and the parasitic patches were kept at a gap of 1 mm. The radiation pattern from the array on the x–z plane was calculated and is shown in Figure 9b. It is worth noting that we achieved a nearly 2 dB better SLL (−18.89 dB) with almost the same level of gain (17.05 dB) compared to the antenna array with the uniformly spaced divider. The non-uniformly spaced power divider also maintains low cross-polarized radiation, 32 dB lower than the co-polarization. A similar bandwidth of 9.64%, compared to the uniformly spaced array from 27.05 to 29.75 GHz, was also achieved, as shown in Figure 9c.

## 3. Fabrication and Measurement 

**Figure 10:**
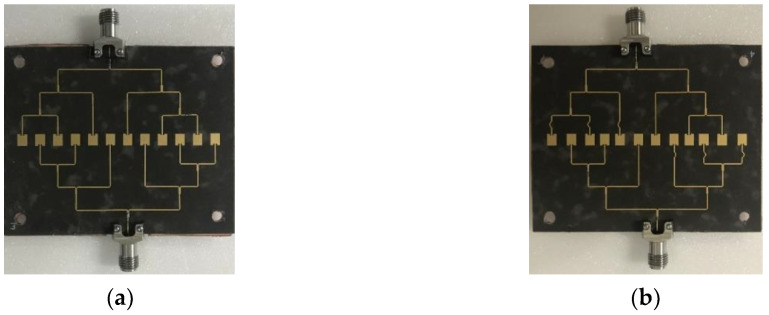
(**a**) Fabricated non-uniformly powered and uniformly spaced antenna array without parasitic patches. (**b**) Fabricated non-uniformly powered and spaced antenna array without parasitic patches.

**Figure 11:**
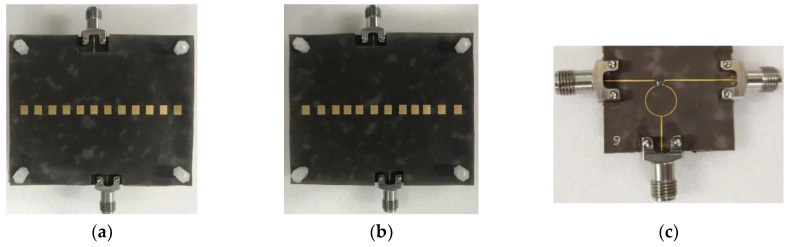
(**a**) Fabricated non-uniformly powered and uniformly spaced antenna array with parasitic patches. (**b**) Fabricated non-uniformly powered and spaced antenna array with parasitic patches. (**c**) Wilkinson power divider for two output ports with 180° phase shift.

**Figure 12:**
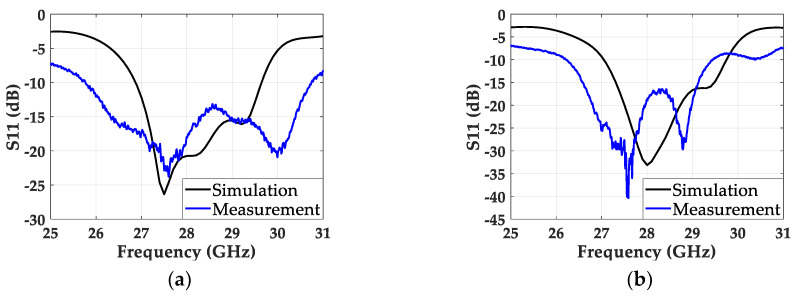
Simulation and measured S_11_ of (**a**) the non-uniformly powered and uniformly spaced antenna array, and (**b**) the non-uniformly powered and spaced antenna array.

For measurement verification, we fabricated the uniformly spaced and the non-uniformly spaced patch antenna arrays in the Rogers 5880 substrate with 70 × 57.3 mm^2^, as shown in Figure 10a,b. Then, the parasitic patches patterned on the same substrate were mounted on top of the array with 1 mm spacing, as depicted in Figure 11a,b. The 1 mm spacing was maintained with four nylon posts on the corners of the antenna array. We also fabricated a Wilkinson power divider to feed two input ports of the antenna array with a 180° phase shift, and show the top view in Figure 11c. The Wilkinson divider was chosen due to the high isolation between the output ports. First, we measured the s-parameters from the Wilkinson power divider and confirmed that two signal paths maintained −6 dB and -6.41 dB attenuation, with a phase difference of 184.2° at 28 GHz. Then, we measured S_11_ from the uniformly and the non-uniformly spaced arrays. Figure 12a,b shows the measured and simulated S_11_ from both arrays, indicating a 15.35% bandwidth (25.16–30.06 GHz) from the uniformly spaced array and an 11.5% bandwidth (26.24–29.46 GHz) from the non-uniformly spaced array. The measured results showed a wider bandwidth than the simulated ones, by nearly 10%; the discrepancy may come from the stronger coupling between the feeding and the parasitic patches due to the higher parasitic effect in the measurement.

**Figure 13:**
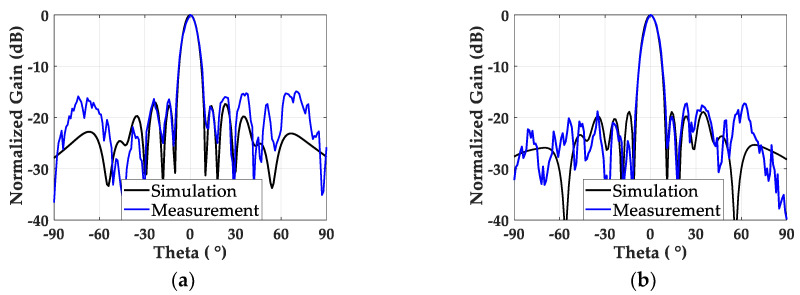
Normalized simulation and measurement gain of (**a**) the non-uniformly powered and uniformly spaced antenna array, and (**b**) the non-uniformly powered and spaced antenna array.

To measure the radiation performance from both antenna arrays, we connected two output ports from the Wilkinson power divider to the two input ports of the antenna array. A horn antenna was used as a transmitting antenna, and a vector network analyzer (Anritsu MS46122B) was used to measure S_21_ between the horn antenna and the designed antenna arrays fed by the Wilkinson power divider. Figure 13a shows the measured normalized gain of the non-uniformly powered and uniformly spaced antenna array. The gain of the antenna array was 16.3 dBi at the boresight, and the first side lobe was found as −15.36 dB with an 8° half-power beamwidth. The measured gain and SLL adhere to the levels from the simulation, with differences of nearly 0.8 dB and 1.5 dB, respectively. The normalized gain of the non-uniformly powered and spaced array from Figure 13b shows a 2 dB lower SLL of −17.27 dB, compared to the uniformly spaced array. This trend follows the simulation results, and experimentally proves the SLL reduction of the non-uniformly spaced patch array operating in the millimeter-wave band. The non-uniformly spaced array also maintained a 16.56 dBi boresight gain and a 7.8° half-power beamwidth, similar to its counterpart.

## 4. Discussion

The measured gain and SLL from both the uniformly and non-uniformly spaced arrays with the non-uniform excitation followed the levels from the simulation results, with a 1.5 dB difference. The differences between the simulation and measurement results can be attributed to the unequal power levels (−6 dB and −6.41 dB) and the non-ideal phase difference (184.2°) between the two output ports of the fabricated Wilkinson power divider. During the radiation measurement, the Wilkinson power divider was mounted on the backside of the antenna array. Therefore, the current flow on the power divider contributed to the elevated radiations, from 55° to 75°, shown in Figure 13. The three-port measurement, which can provide two input signals with the desired phase delay, would be a solution to eliminating the undesired radiation from the extra power divider in the antenna input side. We also expect that fabrication inaccuracy, a possible misalignment of the parasitic patches, and mechanical deformation may contribute to the discrepancy between the simulated and the measured results. The last factor could be the diffraction from the surface wave on the finite antenna ground plane [34]. 

The properties of the reported antenna arrays operating near 28 GHz for 5G applications are summarized in Table 4. We chose the antenna arrays, fed by the microstrip line-based power split network, for a fair comparison. It is worth mentioning that most of the antenna arrays for the 28 GHz band used uniform excitation and spacing, and showed SLL near −13 dB. Only a non-uniformly powered array showed a lower SLL of −15 dB [13]. The proposed microstrip line-based power divider implemented non-uniform inter-element spacing, along with amplitude tapering in the 28 GHz band, for the first time, and demonstrated a superior SLL of −17 dB with the 12-patch antenna array. This SLL is nearly 4 dB lower than the reported −13.4 dB SLL from the parasitic patch-integrated uniformly spaced antenna array [10].

## 5. Conclusions

In this paper, we designed a non-uniformly excited and spaced power divider using the microstrip line to feed a 12-element patch antenna array with parasitic patches for high gain and low SLL at 28 GHz. For higher isolation between adjacent patch antenna elements, including parasitic patches, two signals of input into the power divider with 180° difference were utilized. The fabricated non-uniformly powered and spaced antenna array, integrated with the designed power divider, demonstrated a 16.56 dBi boresight gain and a low SLL of −17.27 dB at 28 GHz. As a counterpart, we also designed a non-uniformly powered but uniformly spaced power divider and the integrated structure with the 12-element patch array showed a higher SLL of −15.3 dB in the measurement. This comparison effectively proved SLL reduction as a result of the non-uniform inter-element spacing. Both antenna arrays also showed a wide bandwidth of more than 10% due to coupling with parasitic patches. Finally, we expect that the non-uniformly powered and spaced high-gain patch antenna array, with a low SLL in the fabrication-friendly microstrip line, can be utilized for a high-gain beam-forming system in millimeter-wave communication.

## Figures and Tables

**Table 1:** Required transmission coefficients, percentages of power, normalized power, and the excitation of the respective ports in the power divider for SLL reduction from Figure 4a.

Port	Transmission Coefficient (dB)	Power (%)	Normalized Power	Normalized Excitation
4	−9	12.5	0.5	0.707
6	−9	12.5	0.5	0.707
8	−6	25	1	1
10	−6	25	1	1
12	−9	12.5	0.5	0.707
14	−9	12.5	0.5	0.707

**Table 2:** Simulated transmission coefficients, percentages of power, normalized power, and the excitation of the respective ports in the power divider for SLL reduction from Figure 4a.

Port	Transmission Coefficient (dB)	Power (%)	Normalized Power	Normalized Excitation
4	−10.22	9.5	0.454	0.673
6	−10.25	9.44	0.45	0.670
8	−6.79	20.94	1	1
10	−6.80	20.89	0.998	0.998
12	−10.23	9.48	0.452	0.672
14	−10.19	9.57	0.457	0.676

**Table 3:** Phase delays from ports 4, 6, 8, 10, 12 and 14 after tuning L1 and L2, and adding the meander lines in the one-to-six power divider, as shown in Figure 7a.

Port	Phase (Degree) Tuning L1 and L2	Phase (Degree) Tuning L1, L2 and Meander Line
4	87.7	86.18
6	87.58	86
8	86.52	85.03
10	98.71	90.12
12	102.11	88.68
14	102.21	89.26

**Table 4:** Performance comparison of the antenna arrays, fed by the microstrip-based power divider, operating near 28 GHz for 5G applications.

Ref#	Power Dist.	Element Spacing	Element No#	fo (GHz)	Gain (dBi)	SLL ^1^ (dB)	Impedance Bandwidth	Radiation Efficiency	Half Power Beam Width
[12]	uniform	uniform	4	28	16.3	−11.6	17.85%	71.8%	11°
[35]	uniform	uniform	8	28	13	~	21.4%	75%	~
[36]	uniform	uniform	8	28	11.32	~	14.1%	~	~
[11]	uniform	uniform	8	28	12	−12.5	17.87%	>65%	12.5°
[13]	non-uniform	uniform	8	26	12	−15	21.15%	>90%	13.3°
[10]	uniform	uniform	16	28	19.66	−13.4	24.4%	86%	5°
This work	non-uniform	uniform	12	28	16.3	−15.36	15.35%	~80%	8.84°
	non-uniform	non-uniform	12	28	16.56	−17.27	11.5%	~80%	8.9°

^1^ Sidelode level (SLL).
